# Who Laughs, and Who Doesn't? Predicting Humor Skills From Personality, Social Anxiety, and Laughter Dispositions of Gelotophobia, Gelotophilia, and Katagelasticism

**DOI:** 10.1111/sjop.70116

**Published:** 2026-05-20

**Authors:** Chloe Lau, Francesca Chiesi, Francesco Bruno, Donald H. Saklofske, Lena C. Quilty

**Affiliations:** ^1^ Temerty Faculty of Medicine University of Toronto Toronto Ontario Canada; ^2^ Centre for Addiction and Mental Health Toronto Ontario Canada; ^3^ Section of Psychology, Department of Neuroscience, Psychology, Drug, and Child's Health (NEUROFARBA) University of Florence Florence Italy; ^4^ Department of Human and Social Sciences, Faculty of Social and Communication Sciences Universitas Mercatorum Rome Italy; ^5^ Department of Psychology University of Western Ontario London Ontario Canada; ^6^ Department of Psychiatry University of Toronto Toronto Ontario Canada

**Keywords:** gelotophobia, humor, laughter, social anxiety, stress

## Abstract

Laughter‐related dispositions, including gelotophobia (fear of being laughed at), gelotophilia (enjoyment of being laughed with), and katagelasticism (enjoyment of laughing at others), may explain patterns of humor use beyond broad personality traits and social anxiety. However, their incremental predictive value across distinct humor domains remains insufficiently examined. A sample of 788 Canadian university students completed self‐report measures of laughter‐related dispositions, HEXACO personality traits, social anxiety, and six humor domains. Hierarchical regression models assessed incremental validity beyond personality and social anxiety. Gradient boosting machine learning models were conducted to examine nonlinear effects and relative predictor importance. Gelotophilia consistently predicted greater humor use across domains. Gelotophobia predicted lower everyday humor, reduced laughing at oneself, and diminished humor under stress, even after controlling for personality and social anxiety. Katagelasticism showed minimal associations with adaptive humor domains. Machine learning analyses converged with regression findings, underscoring the robustness of these effects. Laughter‐related dispositions demonstrate distinct and incremental contributions to humor use beyond personality and social anxiety. Gelotophilia and gelotophobia, in particular, represent meaningful predictors of adaptive humor engagement.

## Key Points

- Gelotophilia consistently emerged as the strongest laughter‐related predictor of humor skills, positively predicting all six humor domains beyond HEXACO personality traits and social anxiety.
- Gelotophobia independently predicted reduced ability to laugh at oneself, lower everyday humor use, and less humor use under stress, supporting its distinct role beyond general social anxiety.
- Katagelasticism showed minimal associations with adaptive humor skills, suggesting that enjoyment of laughing at others may be less relevant to prosocial or self‐regulatory humor domains.
- Hierarchical regression and gradient boosting models converged in identifying gelotophilia and extraversion as robust predictors of humor engagement, while also highlighting context‐specific contributions of gelotophobia, supporting the value of combining traditional and machine learning approaches.

## 1 Introduction

Gelotophobia, defined as the fear of being laughed at, has often been associated with social anxiety but is increasingly recognized as a distinct psychological construct with important unique variance (Ruch and Proyer 2008; Ruch and Proyer 2009; Torres‐Marin et al. 2022). An intriguing theoretical consideration is whether gelotophobia could be conceptualized hierarchically as a narrower‐band construct nested within broader social anxiety, akin to hierarchical models in personality psychology where specific traits reflect shared but also unique variance (Cattell 1946; DeYoung et al. 2007). While both gelotophobia and social anxiety involve fears of negative evaluation and social withdrawal, gelotophobes specifically interpret laughter, regardless of intent, as ridicule (Mattick and Clarke 1998; Proyer and Ruch 2010; Ruch 2009; Ruch et al. 2014). This cognitive bias has been linked to increased amygdala reactivity and heightened attentional sensitivity to social threats (Chang et al. 2025; Hofmann et al. 2017; Platt et al. 2015). Interestingly, Brauer et al. (2022) situated gelotophobia within the maladaptive personality domains of Negative Affectivity, Detachment, and Psychoticism, consistent with prior research linking the construct to Big Five trait equivalents. Gelotophobia was strongly linked to traits reflecting emotional and interpersonal vulnerability, yet showed only modest ties to suspiciousness or unusual beliefs, suggesting it may be a context‐specific disposition centered on sensitivity to laughter and social evaluation (Brauer et al. 2022).

Empirical research continues to support the distinction between gelotophobia and social anxiety. Carretero‐Dios et al. (2010) conducted confirmatory factor analyses demonstrating that gelotophobia, social anxiety, and fear of negative evaluation are best conceptualized as separate constructs. Building on this, Edwards et al. (2010) found that the relationship between gelotophobia and childhood experiences of teasing remained significant even after accounting for social anxiety, reinforcing gelotophobia's unique predictive value. Further evidence from Ritter et al. (2015) showed that attentional and interpretative biases toward laughter are more closely aligned with gelotophobia than with social anxiety alone. Clinical data also reveal that gelotophobia does not uniformly co‐occur with SAD, which further substantiates their empirical differentiation (Havranek, Bolliger, et al. 2017; Havranek, Volkart, et al. 2017).

Torres‐Marin et al. (2022) advanced this line of research using a multitrait–multimethod (MTMM) design incorporating both self‐ and peer assessments. Their results confirmed the discriminant validity of gelotophobia, showing it to be conceptually related to, yet empirically distinct from, both social anxiety and paranoid ideation. Despite these distinctions, gelotophobia and social anxiety share several overlapping traits and correlates, such as introverted neurotic personality profiles, increased anger‐proneness, experiences of bullying, and difficulties with emotion regulation (Ďurka and Ruch 2015; Goldin et al. 2014; Kashdan and McKnight 2010; Kotov et al. 2007; Navarro et al. 2011; Papousek et al. 2014; Proyer et al. 2012; Weiss et al. 2012). These findings underscore the conceptual closeness of the constructs while affirming their empirical separability.

Overall, current research suggests gelotophobia is conceptually distinct from social anxiety, despite some overlap in concerns about negative evaluation (Torres‐Marin et al. 2022). Whereas individuals with social anxiety typically respond to perceived ridicule or embarrassment with avoidance, withdrawal, or behavioral inhibition, individuals high in gelotophobia may show heightened vigilance toward laughter cues and actively seek clarification or reassurance about whether they are being laughed at (Ruch et al. 2014). Thus, rather than uniformly avoiding social situations, gelotophobic individuals may engage in approach‐oriented behaviors aimed at resolving ambiguity about others' laughter (Platt and Forabosco 2012; Ruch 2009). This behavioral pattern underscores that gelotophobia centers specifically on the interpretation of laughter as threatening, rather than reflecting a broader fear of social scrutiny characteristic of social anxiety.

Gelotophilia refers to the enjoyment of being laughed at and reflects a lighthearted, self‐accepting attitude toward humor, even when directed at oneself (Ruch and Proyer 2009). Katagelasticism, in contrast, describes the enjoyment of laughing at others, often at their expense (Ruch and Proyer 2009). These traits are important because they capture distinct social orientations toward humor: gelotophilia is associated with adaptive, prosocial humor use and greater emotional resilience, whereas katagelasticism may relate to more aggressive or socially disruptive humor styles (Ruch and Proyer 2009). Together, they help explain individual differences in how people engage with humor beyond general personality traits, offering insight into both healthy and potentially harmful patterns of humor expression.

The HEXACO model of personality offers a valuable framework for understanding how individual traits relate to both social anxiety and humor expression. High Emotionality, a trait comparable to neuroticism, and low Extraversion have been consistently identified as strong predictors of social anxiety, as they are associated with increased anticipatory anxiety and reduced social engagement (Lee and Ashton 2004; Naragon‐Gainey et al. 2014). Similarly, Torres‐Marín et al. (2019) found distinct personality profiles linked to different laughter‐related dispositions: gelotophobia was associated with lower extraversion, higher emotionality, and reduced honesty‐humility; gelotophilia was related to higher extraversion and openness to experience; and katagelasticism correlated with lower agreeableness and honesty‐humility. Individuals high in gelotophobia often refrain from engaging in positive humor out of fear that it will lead to ridicule, a tendency that has also been connected to reduced life satisfaction (Kuiper et al. 2004; Ruch et al. 2014).

Despite advancements in understanding the interplay between personality traits and humor‐related dispositions, several critical gaps persist. Firstly, the relationship between gelotophobia, the fear of being laughed at, and personality dimensions within the HEXACO model remains underexplored. Particularly, the potential interactions between specific traits and laughter‐related dispositions warrant investigation. For instance, individuals exhibiting low Agreeableness coupled with high Emotionality may be more prone to interpreting laughter as ridicule, intensifying gelotophobic tendencies. Conversely, high levels of Extraversion might serve as a protective factor, mitigating social withdrawal associated with gelotophobia. These potential nonlinear or interactive effects underscore the need for nuanced research in this domain.

Secondly, existing studies have yet to ascertain whether the PhoPhiKat traits, namely gelotophobia, gelotophilia (enjoyment of being laughed with), and katagelasticism (enjoyment of laughing at others), can predict humor behaviors beyond what is explained by HEXACO traits and social anxiety. This gap is particularly salient given the established link between humor‐related traits and well‐being. Notably, the sense of humor has been identified as a malleable construct that can be enhanced through targeted interventions, leading to improved psychological outcomes. For example, the 7 Humor Habits Program has demonstrated efficacy in increasing sense of humor, cheerfulness, and life satisfaction, while concurrently reducing seriousness and negative mood states (McGhee 2010; Ruch et al. 2018). Furthermore, the development and validation of the Short Version of the Sense of Humor Scale (SHS‐S) facilitate the assessment of humor skills training, reinforcing the notion that humor can be cultivated to bolster well‐being (Heintz et al. 2021).

This study seeks to address these gaps by testing whether gelotophobia, gelotophilia, and katagelasticism predict humor use beyond HEXACO and social anxiety, employing both hierarchical regression and boosting regression machine learning to better understand how dispositional fears and joys influence humor expression.

## 2 Methods

### 2.1 Participants

Participants were recruited from a Canadian university psychology participant pool to participate in a study related to personality and humor. Attention checks were placed throughout the survey, and those who failed one or more attention checks or had more than 5% of missing data were removed from the study. The initial sample included 1132 responses, and the final sample included undergraduate students (*N* = 788; 71.4% women; 28.0% men; 0.40% other) enrolled in a large university in Canada. The sample identified as follows: European White (*n* = 326; 41.1%), Asian/Pacific Islander (*n* = 320; 40.7%), Black or African Canadian (*n* = 23; 2.9%), or mixed race/other (15.3%; *n* = 119). Students' ages ranged from 16 to 40 years (*M* = 18.28, SD = 1.29). Participation in the study was voluntary for a credit toward a psychology course, and participants provided informed consent and were debriefed following participation.

### 2.2 Measures

#### 2.2.1 Broad Personality Traits

Participants completed the HEXACO Personality Inventory, which assesses six core personality dimensions using 60 items: Honesty‐Humility (*α* = 0.75, *ω* = 0.75), Emotionality (*α* = 0.74, *ω* = 0.74), Extraversion (*α* = 0.79, *ω* = 0.79), Agreeableness (*α* = 0.75, *ω* = 0.74), Conscientiousness (*α* = 0.79, *ω* = 0.79), and Openness to Experience (*α* = 0.73, *ω* = 0.75). Each trait was measured using self‐report items rated on a Likert scale, with higher scores indicating greater expression of the corresponding trait (Ashton and Lee 2009).

#### 2.2.2 Social Anxiety

Social anxiety was assessed using two well‐validated self‐report measures. The Social Phobia Scale (SPS; *α* = 0.94, *ω* = 0.94) evaluates fear of being observed and scrutinized during performance situations, whereas the Social Interaction Anxiety Scale (SIAS; *α* = 0.94, *ω* = 0.94) assesses anxiety experienced in general social interactions (Mattick and Clark 1998). Higher scores on both scales reflect greater levels of social anxiety.

#### 2.2.3 Laughter Disposition

The PhoPhiKat‐45 is a 45‐item self‐report questionnaire that assesses three distinct dispositions toward laughter and ridicule, using a four‐point Likert scale (Brauer and Proyer 2021; Lau, Li, et al. 2023). The subscales include: gelotophobia (“Pho”; fear of being laughed at; *α* = 0.81, *ω* = 0.82), gelotophilia (“Phi”; enjoyment of being laughed at; *α* = 0.87, *ω* = 0.86), and katagelasticism (“Kat”; enjoyment of laughing at others; *α* = 0.84, *ω* = 0.85). Each subscale demonstrated strong internal consistency, supporting the reliability of the measure.

#### 2.2.4 Sense of Humor (SHS)

Ruch and Heintz (2018) developed the SHS‐Parallel, a 48‐item scale using a seven‐point Likert format, as an updated version of McGhee's (1999) original Sense of Humor Scale. This revised measure demonstrated strong internal consistency and factorial validity across six humor skill dimensions: enjoyment of humor (*α* = 0.73, *ω* = 0.73), laughter (*α* = 0.73, *ω* = 0.73), verbal humor (*α* = 0.86, *ω* = 0.86), finding humor in everyday life (*α* = 0.88, *ω* = 0.88), laughing at oneself (*α* = 0.87, *ω* = 0.87), and humor under stress (*α* = 0.89, *ω* = 0.89). These subscales exhibit strong construct and concurrent validity, reflecting distinct but interrelated components that collectively shape an individual's overall sense of humor (Heintz et al. 2021; Lau et al. 2025). In the present manuscript, we use slightly more descriptive labels for several subscales to improve readability and face‐valid interpretability (e.g., “Everyday Life Humor” for finding humor in everyday life, and “Ability to Laugh at Oneself” for laughing at yourself).

### 2.3 Analytic Approach

Six self‐report measures capturing distinct aspects of SHS were analyzed as dependent variables. For each outcome, a hierarchical multiple regression was conducted, with predictors entered in four sequential blocks. Block 0 (M_0_) includes participant age and sex, block 1 includes HEXACO dimensions (M_1_), block 2 (M_2_) includes social anxiety measures (SPS and SIAS), and block 3 (M_3_) includes laughter disposition scores (gelotophobia, gelotophilia, katagelasticism).

For each model, we examined the total variance explained (*R* ^2^), incremental variance (Δ*R* ^2^) contributed by each block, and conducted *F*‐change tests to assess the significance of these increments. All assumptions for linear regression were checked; no violations were observed, and multicollinearity was minimal (all variance inflation factors < 5.0). Correlations between key variables are found in Table 1. Mean, standard deviation, and all correlations between study variables are found in Supporting Information S1.

**TABLE 1:** Pearson correlations among study variables.

Variable	Pho	Phi	Kat	Honest	Emo	Ex	Agree	Consc	Open	SPS	SIAS
Enjoyment	−0.17***	0.28***	0.03	0.03	0.05*	0.20***	0.17***	0.02	0.08*	−0.09***	−0.16***
Laughter	−0.35***	0.41***	0.06**	0.02	0.46***	0.15***	0.04	0.04	0.23***	−0.32***	−0.15***
Verbal humor	−0.30***	0.53***	0.13***	−0.08*	0.37***	0.09***	0.04	0.13***	0.26***	−0.08*	−0.15***
Everyday life humor	−0.30***	0.47***	0.11***	−0.02	0.44***	0.13***	0.04*	0.00	0.23***	−0.29***	−0.15***
Laugh at self	−0.45***	0.72***	0.09***	−0.09*	0.39***	0.26***	−0.05*	0.01	0.76***	−0.27***	−0.14***
Humor under stress	−0.31***	0.51***	0.18***	−0.15***	0.38***	0.19***	−0.06	0.04	0.55***	−0.25***	−0.15***

*Note:* Pearson's *r* coefficients are reported.

Abbreviations: Agree = agreeableness, Consc = conscientiousness, Emo = emotionality, Ex = extraversion, Honest = honesty‐humility, Kat = katagelasticism, Open = openness to experience, Phi = gelotophilia, Pho = gelotophobia, SIAS = Social Interaction Anxiety Scale, SPS = Social Phobia Scale.

* *p* < 0.05.

** *p* < 0.01.

*** *p* < 0.001.

To complement the linear regression analyses, gradient boosting regression models were fitted for each SHS outcome variable. Gradient boosting is an ensemble learning method that sequentially builds decision trees, with each tree trained to reduce the residual error of the preceding model by minimizing a specified loss function (here, least‐squares loss). Data were randomly partitioned into training (60%), validation (20%), and test (20%) subsets. Model tuning and early stopping were guided by validation set performance to reduce overfitting. Final model performance was evaluated using mean squared error (MSE) on the held‐out test set.

All predictors included in the linear regression models were entered as features in the boosting analyses. Because gradient boosting can flexibly model nonlinear relationships and higher‐order interactions without prespecification, it provides a complementary, assumption‐light approach to examining predictive structure.

Feature importance was assessed using (a) relative importance metrics derived from the boosting algorithm and (b) permutation‐based importance, quantified as the mean increase in test‐set MSE following random permutation of each predictor (50 repetitions). These importance estimates were compared with regression coefficients and statistical significance patterns from the linear models to evaluate convergence across analytic frameworks and to identify robust predictors. All analyses were conducted in JASP (Version 0.19.2). Given the exploratory and comparative nature of the boosting analyses, alpha corrections were not applied to the linear regression models in order to facilitate descriptive cross‐method comparisons.

## 3 Results

Tables 2 and 3 describe a summary of findings. Additional details for all regression and boosting machine learning regression values are found in Supporting Information S2 and S3.

**TABLE 2:** Standardized beta coefficients for predictors of humor outcomes in hierarchical regression models (model 3).

Predictor	Laughter	Laughing at self	Humor under stress	Verbal humor	Everyday humor	Humor enjoyment
Gender	−0.01	0.01	−0.02	0.01	0	0
Age	−0.04	−0.05	−0.03	−0.02	−0.02	−0.01
Honesty	−0.01	0.05	−0.02	−0.04	−0.05	−0.01
Emotionality	0.11**	0.03	−0.05	−0.01	0.06	0.12*
Extraversion	0.30**	0.08*	0.19**	0.15**	0.26**	0.05
Agreeableness	0.04	0.07*	0.10*	−0.02	0.06	0.12*
Conscientiousness	−0.03	−0.04	−0.02	0.08*	0.06	0.06
Openness	0.04	0.01	0.07	0.15**	0.10*	0.07*
SPS	0.05	0.03	0.17*	0.11*	0.05	0.09
SIAS	−0.07	0.06	−0.09	−0.09	0.01	−0.14*
Pho	−0.08	−0.24**	−0.11*	−0.08	−0.13*	−0.03
Phi	0.27**	0.66**	0.37**	0.45**	0.36**	0.23**
Kat	0.01	−0.04	0.07	0.05	0	0.03
M0 R2	0	0	0	0	0	0
M1 R2	0.23**	0.22***	0.19***	0.16***	0.22***	0.07***
M2 R2	0.23***	0.22***	0.19***	0.16***	0.22***	0.08***
M3 R2	0.30***	0.59***	0.33***	0.35***	0.34***	0.12***

*Note:* M_0_ includes Gender, Age. M_1_ includes M_0_ with Honesty, Emotionality, Extraversion, Agreeableness, Conscientiousness, Openness. M_2_ includes M_1_ with SPS, SIAS. M_3_ includes M_2_ with Pho, Phi, Kat. Significance for standardized Beta coefficients includes *p* < 0.05*, *p* < 0.01**, *p* < 0.001***.

Abbreviations: Kat = Katagelasticism, Phi = Gelotophilia, Pho = Gelotophobia, SIAS = Social Interaction Anxiety Scale, SPS = Social Phobia Scale.

**TABLE 3:** Relative importance values of the boosting regression machine learning model.

Predictor	Enjoyment of humor	Laughter	Verbal humor	Everyday humor	Laughing at self	Humor under stress
Phi	35.58	18.97	66.38	42.88	76.41	58.79
Pho	8.99	1.85	10.63	22.41	17.39	1.57
Kat	0	0	1.05	0	0	1.64
Extraversion	18.17	57.92	9.08	26.57	2.07	22.73
Agreeableness	25.56	0	0.84	4.89	3.12	11.73
Openness	4.95	2	7.29	1.78	0	0
Honesty	0	0	1.38	0	1	0
Conscientiousness	0	1.48	1.91	0	0	0
Emotionality	0	3.15	0	1.47	0	0
Age	0	2.43	0	0	0	0
Gender	0	0	0	0	0	0
SPS	6.74	0	0	0	0	0
SIAS	0	12.21	1.44	0	0	3.54

Abbreviations: Kat = Katagelasticism, Phi = Gelotophilia, Pho = Gelotophobia, SIAS = Social Interaction Anxiety Scale, SPS = Social Phobia Scale.

### 3.1 Laughter

Regression analysis of general laughter frequency revealed that demographic variables explained minimal variance (*R* ^2^ < 0.01). The inclusion of personality traits in M_1_ significantly improved the model (total *R* ^2^ = 0.23). Adding social anxiety measures in M_2_ significantly contributed further, although the total explained variance remained stable (*R* ^2^ = 0.23). M_3_ included laughter‐related dispositions from the PhoPhiKat, accounted for approximately 30% of the variance in laughter frequency (Δ*R* ^2^ = 0.07, *p* < 0.001). In this model, extraversion emerged as the strongest predictor (*β* = 0.30, *p* < 0.001), followed by gelotophilia (*β* = 0.27, *t* = 7.46, *p* < 0.001). Emotionality also remained a significant positive predictor (*β* = 0.11, *p* = 0.001).

Gradient boosting regression showed modest predictive performance for laughter frequency (test‐set MSE = 0.64; test‐set *R* ^2^ = 0.18) based on a 499/125/155 train–validation–test split. Extraversion was the most influential predictor (RI = 57.92; permutation importance [mean dropout loss] = 0.84), followed by gelotophilia (RI = 18.97; 0.76), SIAS (RI = 12.21; 0.74), and emotionality (RI = 3.15; 0.73). Although SIAS did not emerge as a significant predictor in the linear regression model, it showed notable importance in the boosting model, suggesting it may contribute via nonlinear and/or interaction effects not captured by the additive linear specification. Full details for all models are provided in Supporting Information S2 and S3.

### 3.2 Ability to Laugh at Oneself

Hierarchical regression predicting the ability to laugh at oneself demonstrated that the inclusion of personality traits and social anxiety measures in M_1_ and M_2_, respectively, significantly improved model fit (Δ*R* ^2^ = 0.22, *p* < 0.001). The addition of laughter‐related dispositions in M_3_ further enhanced prediction (Δ*R* ^2^ = 0.37, *p* < 0.001), bringing the total explained variance to *R* ^2^ = 0.59. In the final model, gelotophobia was a strong negative predictor of the ability to laugh at oneself (*β* = −0.24, *p* < 0.001), while gelotophilia emerged as a robust positive predictor (*β* = 0.66, *p* < 0.001). Extraversion (*β* = 0.08, *p* < 0.01) and agreeableness (*β* = 0.07, *p* < 0.01) also positively predicted laughing at oneself. Other personality traits were not significant.

Gradient boosting regression for laughing at oneself yielded a test‐set MSE of 0.54 (*R* ^2^ = 0.56). Gelotophilia emerged as the dominant predictor (RI = 76.41; permutation importance = 1.12), followed by gelotophobia (RI = 17.39; 0.77), agreeableness (RI = 3.12; 0.71), and extraversion (RI = 2.07; 0.70). All remaining predictors showed negligible relative influence. The boosting model therefore highlighted gelotophilia and gelotophobia as primary drivers of the ability to laugh at oneself, with personality traits contributing more modestly. This pattern was broadly consistent with the linear regression results, which similarly identified gelotophilia and gelotophobia as key predictors.

### 3.3 Humor Use Under Stress

Hierarchical regression predicting humor use under stress showed M_1_ and M_2_ significantly improved the model (Δ*R* ^2^ = 0.19, *p* < 0.001). Laughter dispositions (M_3_) further improved prediction modestly (Δ*R* ^2^ = 0.14, total *R* ^2^ = 0.33, *p* < 0.001). In the final model, gelotophilia remained the strongest predictor (*β* = 0.37, *p* < 0.001), and extraversion remained a significant positive predictor (*β* = 0.19, *p* < 0.001) with agreeableness (*β* = 0.10, *p* < 0.01). Gelotophobia showed a negative effect (*β* = −0.11, *p* < 0.05).

Gradient boosting regression for humor under stress yielded a test‐set MSE of 0.95 (*R* ^2^ = 0.20). Gelotophilia emerged as the most influential predictor (RI = 58.79; permutation importance = 1.03), followed by extraversion (RI = 22.73; permutation importance = 0.94), agreeableness (RI = 11.73; permutation importance = 0.91), and SIAS (RI = 3.54; permutation importance = 0.89). All remaining predictors contributed minimally to model performance. The convergence between the boosting and linear models underscores the central role of gelotophilia and extraversion in humor use under stress, while also highlighting the added explanatory contribution of agreeableness and stress‐related coping tendencies in more complex predictive structures.

### 3.4 Everyday Life Humor

Hierarchical regression predicting everyday life humor revealed that the inclusion of personality traits and social anxiety in M_1_ and M_2_ significantly increased the explained variance (Δ*R* ^2^ = 0.22, *p* < 0.001). The addition of laughter dispositions in M_3_ further enhanced the model (ΔR^2^ = 0.12, *p* < 0.001). In the final model, gelotophilia emerged as the strongest positive predictor (*β* = 0.36, *p* < 0.001), followed by extraversion (*β* = 0.26, *p* < 0.001). Gelotophobia negatively predicted everyday humor use (*β* = −0.13, *p* < 0.01).

Gradient boosting regression for everyday humor use yielded a test‐set MSE of 0.59 (*R* ^2^ = 0.23), closely aligning with the linear model's performance. Gelotophilia emerged as the most influential predictor (RI = 42.88; permutation importance = 0.88), followed by extraversion (RI = 26.57; permutation importance = 0.82) and gelotophobia (RI = 22.41; permutation importance = 0.82). Agreeableness, openness, and emotionality contributed to a lesser extent, whereas remaining predictors showed minimal influence. The convergence between boosting and linear regression findings suggests that everyday humor use is largely driven by the additive effects of laughter‐related dispositions and extraversion, with limited evidence that a more complex nonlinear structure substantially improves prediction.

### 3.5 Verbal Humor Ability

The inclusion of personality traits and social anxiety measures in M_1_ and M_2_, respectively, significantly improved the model predicting verbal humor ability (*R* ^2^ = 0.16, *p* < 0.001). The addition of laughter dispositions in M_3_ further increased the explained variance (*R* ^2^ = 0.35, *p* < 0.001). In the final model, gelotophilia emerged as the strongest predictor (*β* = 0.45, *p* < 0.001), followed by extraversion (*β* = 0.15, *p* < 0.001), openness to experience (*β* = 0.15, *p* < 0.001), conscientiousness (*β* = 0.08, *p* < 0.05), and social anxiety on the Social Phobia Scale (SPS; *β* = 0.11, *p* < 0.05). Gradient boosting regression for verbal humor yielded a test‐set MSE of 0.89 (*R* ^2^ = 0.34). Gelotophilia emerged as the dominant predictor (RI = 66.38; permutation importance = 0.97), followed by gelotophobia (RI = 10.63; permutation importance = 0.79) and extraversion (RI = 9.08; permutation importance = 0.79). All remaining predictors contributed minimally to model performance.

### 3.6 Enjoyment of Humor

Hierarchical regression predicting humor enjoyment explained approximately 12% of the variance. Demographic variables contributed negligibly to the model (*R* ^2^ < 0.01). The addition of personality traits in Model 1 accounted for a moderate increase in explained variance (Δ*R* ^2^ = 0.07, *p* < 0.001), while Model 2, which added social anxiety measures, yielded a modest but significant increase (Δ*R* ^2^ = 0.01, *p* < 0.05). Model 3, which introduced laughter dispositions, further modestly improved prediction (final *R* ^2^ = 0.12, *p* < 0.001). Within the final model, gelotophilia was positively associated with humor enjoyment (*β* = 0.23, *p* < 0.001). Emotionality (*β* = 0.12, *p* < 0.01), openness to experience (*β* = 0.07, *p* < 0.05), and SIAS scores (*β* = −0.14, *p* < 0.05) were also significant predictors.

Complementary gradient boosting regression for humor enjoyment yielded a test‐set MSE of 0.81 (*R* ^2^ = 0.08), indicating modest predictive performance. Gelotophilia emerged as the most influential predictor (RI = 35.58; permutation importance = 0.85). Agreeableness (RI = 25.56; permutation importance = 0.84) and extraversion (RI = 18.17; permutation importance = 0.84) also contributed meaningfully. Gelotophobia (RI = 8.99) and SPS (RI = 6.74) showed smaller but nontrivial contributions. All remaining predictors, including katagelasticism and other HEXACO traits, demonstrated negligible influence. The similarity between boosting and linear model performance suggests limited additional predictive benefit from more complex nonlinear structure.

## 4 Discussion

This study examined how broad personality traits, social anxiety dimensions, and laughter‐related dispositions, namely gelotophobia, gelotophilia, and katagelasticism, uniquely and collectively predict humor appreciation and use across various domains. The findings support and extend existing literature by demonstrating that although gelotophobia shares conceptual overlap with social anxiety, it operates as a distinct construct with specific negative associations with humor‐related behaviors (Ruch et al. 2014; Torres‐Marin et al. 2022). Hierarchical regression analyses showed that gelotophobia uniquely predicted reduced capacity to laugh at oneself, lower humor use under stress, and diminished everyday humor, even when controlling for HEXACO traits and social anxiety. These findings align with previous research (e.g., Ruch et al. 2014; Torres‐Marin et al. 2022) and reinforce gelotophobia's inhibitory role in humor expression, particularly in self‐referential and socially demanding contexts. Notably, this pattern appears to evolve with age: while gelotophobia is not associated with humor coping in adolescence (Führ et al. 2015), it is negatively linked to such strategies in adulthood (Ruch and Proyer 2009; Ruch et al. 2014).

In contrast, gelotophilia consistently emerged as one of the strongest positive predictors of humor across all dimensions, second only to extraversion in predicting laughter frequency. This replicates and builds on prior findings connecting gelotophilia to humor engagement and creative expression (Lau et al. 2022; Lau et al. 2024; Ruch et al. 2014). Gelotophilia reflects a positive, open attitude toward being laughed at, which likely fosters emotional resilience and social ease (Ruch and Proyer 2009).

Extraversion and emotionality also showed robust associations with humor. In line with previous work (Lee and Ashton 2004; Naragon‐Gainey et al. 2014), extraversion was linked to a wide range of humor skills, including overall laughter frequency, verbal humor, and the ability to laugh at oneself, likely due to increased sociability and reduced anticipatory anxiety. Emotionality positively predicted humor enjoyment and frequency, potentially reflecting greater affective responsiveness to humorous stimuli. Additionally, openness was a significant predictor of verbal humor, suggesting that this humor domain may align more closely with ability‐based humor rather than trait‐like or habitual expressions. Certain forms of humor may rely more on creativity and cognitive flexibility than on general tendencies to engage with humor (Ruch and Heintz 2019).

Gradient boosting regression models provided additional insights, supporting the central role of gelotophilia and extraversion while also highlighting potentially more complex or non‐linear predictive relationships. Notably, SIAS emerged as an important predictor in the machine learning models, despite showing weaker effects in the linear regressions. This discrepancy suggests that social anxiety may interact with other traits or exhibit non‐linear influences on humor behaviors that are better captured by ensemble models. These findings highlight the value of combining traditional and machine learning approaches in psychological research.

### 4.1 Limitations and Future Directions

Several limitations warrant consideration. The exclusive use of self‐report measures may introduce biases due to social desirability or limited self‐awareness. Future studies should incorporate peer reports or control for social desirability to validate and extend these findings (e.g., Lau, Li, et al. 2023). Moreover, although katagelasticism was not a strong predictor of adaptive humor use in this study, prior research suggests its relevance in other domains. For example, it has been shown to incrementally predict online trolling behavior even when controlling for gender and dark personality traits (Navarro‐Carrillo et al. 2021). Future research should explore the role of katagelasticism for more antagonistic types of humor.

Although we note that our sample characteristics may constrain generalizability, we wish to clarify that many associations in personality and humor research obtained in student populations replicate in more heterogeneous samples. The more critical issue is whether the *distribution* of key constructs in a given sample, including means and variances, adequately approximates the broader population. In particular, student samples may exhibit restriction of range for certain traits or dispositions (e.g., attenuated variability in openness or other relevant personality dimensions), which can bias variance estimates, reduce statistical power, and attenuate observed associations. Accordingly, future replications would benefit from sampling strategies that explicitly target broader distributions of the focal traits and humor‐related dispositions, allowing stronger inferences about effect sizes across the full latent continuum.

A second, related direction concerns assessment modality. Humor research relies heavily on questionnaires, and the present study is no exception (Martin et al. 2003). While self‐report measures are well suited for capturing stable dispositions and subjective experiences, they do not directly index humor behavior as it unfolds in social interaction. At the same time, we acknowledge that isolated behavioral “single acts” can be unreliable and may not provide sufficient differentiation across individuals. Future work could therefore adopt multi‐method designs that combine trait measures with repeated behavioral indicators across contexts (e.g., experience‐sampling reports of humor episodes, informant ratings, or standardized interaction tasks coded across multiple occasions). Such approaches would allow stronger tests of whether laughter‐related dispositions predict observable humor behavior and interpersonal outcomes, while improving reliability through aggregation across situations and time.

An important conceptual limitation of the present study concerns the “virtue gap” in humor research (Ruch and Heintz 2016). The McGhee Humor Skills Scale (SHS) primarily assesses humor competence, flexibility, and adaptive use of humor for social–emotional regulation, but does not directly capture morally valenced forms of humor such as benevolent, corrective, or virtue‐based humor. Thus, our operationalization of humor skills focuses on technical and regulatory aspects of humor rather than moral orientation. In this respect, the SHS is largely orthogonal to constructs of benevolence and malevolence, which may help explain why Honest‐Humility did not emerge as a strong predictor in the present analyses. Future research aiming to bridge the virtue gap would benefit from integrating measures of virtuous humor grounded in the HEXACO framework, particularly the Honest‐Humility dimension, to more directly assess humor “coming from the heart” or reflecting prosocial moral intent. Such work would extend beyond competence‐based humor skills and provide a more comprehensive understanding of the moral and characterological dimensions of humor.

An important direction for future research concerns the role of laughter‐related dispositions within romantic relationships. Prior work has demonstrated that smiling and laughter are central interpersonal signals that communicate positive affect, romantic interest, and relational intent (Algoe 2019; Montoya et al. 2018). Moreover, dispositions toward ridicule and being laughed at have been linked to attachment styles, relationship status, and relationship satisfaction (Brauer and Proyer 2018, 2020; Brauer et al. 2020). For example, gelotophobia has been associated with attachment insecurity and a greater likelihood of remaining single, suggesting that heightened fear of being laughed at may interfere with intimacy formation. Building on this literature, future research could examine a serial pathway in which laughter‐related dispositions (gelotophobia, gelotophilia, katagelasticism) influence humor behaviors, which in turn shape romantic outcomes such as attachment dynamics, relationship initiation, satisfaction, and stability. Such a model would integrate personality‐based dispositions toward laughter with observable humor behaviors and downstream relational functioning, thereby embedding the study of laughter within a broader interpersonal framework. Examining these pathways longitudinally would further clarify whether humor behaviors mediate the association between laughter‐related dispositions and relationship trajectories over time.

## 5 Conclusion

In sum, this study provides robust empirical evidence for the distinct roles of laughter‐related dispositions associated with humor behavior. Gelotophobia and gelotophilia emerged as particularly salient predictors, with gelotophilia consistently associated with greater humor use and appreciation. Findings support the differentiation between gelotophobia and social anxiety disorder and emphasize the importance of incorporating laughter dispositions into both personality assessment and clinical evaluation. Recognizing these traits may offer valuable insights into social functioning, emotional resilience, and the therapeutic potential of humor.

## Author Contributions

Chloe Lau contributed to study conceptualization, data collection, data preparation, data analysis, report writing. Francesca Chiesi and Francesco Bruno contributed to data analysis and report writing. Donald H. Saklofske contributed to supervision, data collection and data preparation. Lena C. Quilty contributed to study conceptualization, report writing, and supervision. All authors have read, edits, and approved the manuscript prior to submission.

## Funding

The authors have nothing to report.

## Disclosure

Preregistration statement: The study was not pre‐registered.

## Ethics Statement

The study was approved by the Western University Research Ethics Board. All procedures performed in studies involving human participants were in accordance with the ethical standards of Canadian national and international guidelines as well as provincial legislation and hospital policies and with the 1964 Helsinki Declaration and its later amendments or comparable ethical standards.

## Consent

This study was conducted according to the American Psychological Association (APA) and National Association of Psychology ethical standards for the treatment of human subjects. Identifying information was separated from the dataset upon analysis. Participants were informed that their participation was voluntary, that they could leave the study at any time, and that their data would be treated anonymously. Oral and written informed consent was obtained and documented by a research team member.

## Conflicts of Interest

The authors declare no conflicts of interest.

## Supporting information


**Data S1:** sjop70116‐sup‐0001‐DataS1.docx.

**Data S2:** sjop70116‐sup‐0002‐DataS2.docx.

**Data S3:** sjop70116‐sup‐0003‐DataS3.docx.

## Data Availability

The data supporting the findings of this study are not publicly available, as data sharing was not included in the original ethics application, and participants were not informed that their anonymized data might be shared online. Data can be made available from the authors upon reasonable request, in accordance with institutional and ethical guidelines.
