# Supplementary material for: Who Laughs, and Who Doesn't? Predicting Humor Skills From Personality, Social Anxiety, and Laughter Dispositions of Gelotophobia, Gelotophilia, and Katagelasticism
Source: Scand J Psychol. 2026 May 20;67(6):1382–91. doi: 10.1111/sjop.70116 (PMC13739008; doi:10.1111/sjop.70116)
Supplement: Supplementary file 1 [file SJOP-67-1382-s001.docx]

# Supplementary 3: Boosting Regression Machine Learning

# Table 1 Boosting Regression Results: Enjoyment of Humor

## Model Performance Metrics

| Metric | Value |
| --- | --- |
| MSE | 0.814 |
| MSE (scaled) | 1.439 |
| RMSE | 0.902 |
| MAE / MAD | 0.705 |
| MAPE | 17.48% |
| R² | 0.076 |

## Feature Importance Metrics

| Feature | Relative Influence | Mean Dropout Loss |
| --- | --- | --- |
| Phi | 35.58267 | 0.85187 |
| Agreeableness | 25.56391 | 0.84145 |
| Extraversion | 18.17443 | 0.83715 |
| Pho | 8.98695 | 0.83318 |
| SPS | 6.73843 | 0.83235 |
| Openness | 4.95360 | 0.83193 |
| Age | 0.00000 | 0.82984 |
| Gender | 0.00000 | 0.82984 |
| Kat | 0.00000 | 0.82984 |
| Honesty | 0.00000 | 0.82984 |
| Emotionality | 0.00000 | 0.82984 |
| Conscientiousness | 0.00000 | 0.82984 |
| SISS | 0.00000 | 0.82984 |

# Table 2 Boosting Regression Results: Predicting Laughter Frequency

## Model Performance Metrics

| Metric | Value |
| --- | --- |
| MSE | 0.638 |
| MSE (scaled) | 1.152 |
| RMSE | 0.799 |
| MAE / MAD | 0.612 |
| MAPE | 14.35% |
| R² | 0.176 |

## Feature Importance Metrics

| Feature | Relative Influence | Mean Dropout Loss |
| --- | --- | --- |
| Extraversion | 57.91845 | 0.8358 |
| Phi | 18.96623 | 0.76421 |
| SISS | 12.21173 | 0.73962 |
| Emotionality | 3.14551 | 0.73414 |
| Age | 2.43334 | 0.73074 |
| Openness | 2.00014 | 0.73123 |
| Pho | 1.84608 | 0.73080 |
| Conscientiousness | 1.47852 | 0.73095 |
| Gender | 0.00000 | 0.72898 |
| Kat | 0.00000 | 0.72898 |
| Honesty | 0.00000 | 0.72898 |
| Agreeableness | 0.00000 | 0.72898 |
| SPS | 0.00000 | 0.72898 |

# Table 3 Boosting Regression Results: Predicting Verbal Humor

## Model Performance Metrics

| Metric | Value |
| --- | --- |
| MSE | 0.886 |
| MSE (scaled) | 0.836 |
| RMSE | 0.941 |
| MAE / MAD | 0.725 |
| MAPE | 19.62% |
| R² | 0.335 |

## Feature Importance Metrics

| Feature | Relative Influence | Mean Dropout Loss |
| --- | --- | --- |
| Phi | 66.37828 | 0.96710 |
| Pho | 10.63312 | 0.79238 |
| Extraversion | 9.07825 | 0.79120 |
| Openness | 7.28891 | 0.78853 |
| Conscientiousness | 1.91026 | 0.78021 |
| SISS | 1.44311 | 0.77949 |
| Honesty | 1.37798 | 0.77969 |
| Kat | 1.04703 | 0.77792 |
| Agreeableness | 0.84306 | 0.77807 |
| Age | 0.00000 | 0.77709 |
| Gender | 0.00000 | 0.77709 |
| Emotionality | 0.00000 | 0.77709 |
| SPS | 0.00000 | 0.77709 |

# Table 4 Boosting Regression Results for Predicting Everyday Life Humor

Model Performance Metrics

| Metric | Value |
| --- | --- |
| MSE | 0.595 |
| MSE (scaled) | 1.036 |
| RMSE | 0.771 |
| MAE / MAD | 0.604 |
| MAPE | 12.86% |
| R² | 0.229 |

Feature Importance Metrics

| Feature | Relative Influence | Mean Dropout Loss |
| --- | --- | --- |
| Phi | 42.87861 | 0.87575 |
| Extraversion | 26.57204 | 0.82496 |
| Pho | 22.40978 | 0.81710 |
| Agreeableness | 4.89096 | 0.79162 |
| Openness | 1.77964 | 0.78510 |
| Emotionality | 1.46896 | 0.78452 |
| Age | 0.00000 | 0.78310 |
| Gender | 0.00000 | 0.78310 |
| Kat | 0.00000 | 0.78310 |
| Honesty | 0.00000 | 0.78310 |
| Conscientiousness | 0.00000 | 0.78310 |
| SPS | 0.00000 | 0.78310 |
| SISS | 0.00000 | 0.78310 |

Note. Mean dropout loss (defined as root mean squared error [RMSE]) is based on 50 permutations.

# Table 5 Boosting Regression Predicting Laughing at Oneself

Model Performance Metrics

| Metric | Value |
| --- | --- |
| MSE | 0.541 |
| MSE (scaled) | 0.504 |
| RMSE | 0.736 |
| MAE / MAD | 0.535 |
| MAPE | 15.41% |
| R² | 0.557 |

Feature Importance Metrics: Relative Influence

| Feature | Relative Influence | Mean Dropout Loss |
| --- | --- | --- |
| Phi | 76.41 | 1.12271 |
| Pho | 17.39 | 0.76977 |
| Agreeableness | 3.12 | 0.70530 |
| Extraversion | 2.07 | 0.69952 |
| Honesty | 1.00 | 0.69768 |
| Age | 0.00 | 0.69543 |
| Gender | 0.00 | 0.69543 |
| Kat | 0.00 | 0.69543 |
| Emotionality | 0.00 | 0.69543 |
| Conscientiousness | 0.00 | 0.69543 |
| Openness | 0.00 | 0.69543 |
| SPS | 0.00 | 0.69543 |
| SISS | 0.00 | 0.69543 |

# Table 6

# Boosting Regression Predicting Humor Under Stress

# Model Performance Metrics

| MSE | 0.946 |
| --- | --- |
| MSE(scaled) | 1.1 |
| RMSE | 0.973 |
| MAE / MAD | 0.740 |
| MAPE | 20.54% |
| R² | 0.199 |

# Feature Importance Metrics

| Variable | Relative Influence | Mean Dropout Loss |
| --- | --- | --- |
| Phi | 58.79312 | 1.02821 |
| Extraversion | 22.72794 | 0.93780 |
| Agreeableness | 11.72836 | 0.91458 |
| SISS | 3.54141 | 0.89285 |
| Kat | 1.64306 | 0.89137 |
| Pho | 1.56612 | 0.89133 |
| Age | 0.00000 | 0.88901 |
| Gender | 0.00000 | 0.88901 |
| Honesty | 0.00000 | 0.88901 |
| Emotionality | 0.00000 | 0.88901 |
| Conscientiousness | 0.00000 | 0.88901 |
| Openness | 0.00000 | 0.88901 |
| SPS | 0.00000 | 0.88901 |
