# Supplementary material for: Who Laughs, and Who Doesn't? Predicting Humor Skills From Personality, Social Anxiety, and Laughter Dispositions of Gelotophobia, Gelotophilia, and Katagelasticism
Source: Scand J Psychol. 2026 May 20;67(6):1382–91. doi: 10.1111/sjop.70116 (PMC13739008; doi:10.1111/sjop.70116)
Supplement: Supplementary file 2 [file SJOP-67-1382-s002.docx]

Supplementary Materials 1 .

| *Descriptive Statistics* | | | | | | | | | | | | | | | | | |
| --- | --- | --- | --- | --- | --- | --- | --- | --- | --- | --- | --- | --- | --- | --- | --- | --- | --- |
|  | | SPS | | SISS | | Enjoyment | | Laughter | | VerbalHumor | | EverydayLifeHum | | LaughSelf | | HumStress | |
| Valid |  | 788 |  | 788 |  | 787 |  | 787 |  | 786 |  | 787 |  | 788 |  | 788 |  |
| Missing |  | 0 |  | 0 |  | 1 |  | 1 |  | 2 |  | 1 |  | 0 |  | 0 |  |
| Mean |  | 2.278 |  | 2.381 |  | 4.858 |  | 4.867 |  | 4.970 |  | 5.258 |  | 4.809 |  | 4.752 |  |
| Std. Deviation |  | 0.786 |  | 0.784 |  | 0.888 |  | 0.869 |  | 0.988 |  | 0.917 |  | 1.065 |  | 1.082 |  |
| Minimum |  | 1.000 |  | 1.000 |  | 1.500 |  | 1.750 |  | 1.125 |  | 1.875 |  | 1.125 |  | 1.250 |  |
| Maximum |  | 5.000 |  | 5.000 |  | 7.000 |  | 6.875 |  | 7.000 |  | 7.000 |  | 7.000 |  | 7.000 |  |
|  | | | | | | | | | | | | | | | | | |

| *Descriptive Statistics* | | | | | | | | | | | | | | | | | | | |
| --- | --- | --- | --- | --- | --- | --- | --- | --- | --- | --- | --- | --- | --- | --- | --- | --- | --- | --- | --- |
|  | | Pho | | Phi | | Kat | | Honesty | | Emotionality | | Extraversion | | Agreeableness | | Conscientiousness | | Openness | |
| Valid |  | 785 |  | 785 |  | 783 |  | 788 |  | 787 |  | 787 |  | 786 |  | 787 |  | 788 |  |
| Missing |  | 3 |  | 3 |  | 5 |  | 0 |  | 1 |  | 1 |  | 2 |  | 1 |  | 0 |  |
| Mean |  | 2.316 |  | 2.532 |  | 2.190 |  | 3.099 |  | 3.499 |  | 3.331 |  | 3.168 |  | 3.670 |  | 3.229 |  |
| Std. Deviation |  | 0.452 |  | 0.491 |  | 0.473 |  | 0.628 |  | 0.601 |  | 0.625 |  | 0.582 |  | 0.592 |  | 0.627 |  |
| Minimum |  | 1.000 |  | 1.200 |  | 1.000 |  | 1.100 |  | 1.500 |  | 1.100 |  | 1.300 |  | 1.800 |  | 1.300 |  |
| Maximum |  | 3.733 |  | 3.867 |  | 3.733 |  | 4.900 |  | 5.000 |  | 5.000 |  | 5.000 |  | 4.900 |  | 4.800 |  |

| Full Correlation Table.  Pearson Correlations Among Study Variables (Rounded to Two Decimal Places) |  |  |
| --- | --- | --- |

| Variable | 1. Pho | 2. Phi | 3. Kat | 4. Honesty | 5. Emotionality | 6. Extraversion | 7. Agreeableness | 8. Conscientiousness | 9. Openness | 10. SPS | 11. SIAS | 12. Enjoyment | 13. Laughter | 14. VerbalHumor | 15. EverydayLifeHum | 16. LaughSelf |
| --- | --- | --- | --- | --- | --- | --- | --- | --- | --- | --- | --- | --- | --- | --- | --- | --- |
| 1. Pho | — |  |  |  |  |  |  |  |  |  |  |  |  |  |  |  |
| 2. Phi | -.33*** | — |  |  |  |  |  |  |  |  |  |  |  |  |  |  |
| 3. Kat | .15*** | .31*** | — |  |  |  |  |  |  |  |  |  |  |  |  |  |
| 4. Honesty | -.14*** | .01 | -.40*** | — |  |  |  |  |  |  |  |  |  |  |  |  |
| 5. Emotionality | .20*** | -.14*** | -.29*** | .09 | — |  |  |  |  |  |  |  |  |  |  |  |
| 6. Extraversion | -.59*** | .36*** | .03 | -.03 | -.10** | — |  |  |  |  |  |  |  |  |  |  |
| 7. Agreeableness | -.23*** | -.17*** | -.24*** | .33*** | -.09** | .15*** | — |  |  |  |  |  |  |  |  |  |
| 8. Conscientiousness | -.15*** | -.12*** | -.27*** | .17*** | .21*** | .12*** | .01 | — |  |  |  |  |  |  |  |  |
| 9. Openness | -.06 | -.05 | -.16** | .04 | .02 | .13*** | .07* | -.03 | — |  |  |  |  |  |  |  |
| 10. SPS | -.67*** | -.17*** | -.19*** | -.26*** | -.48*** | -.13*** | -.12*** | -.03 | .39*** | — |  |  |  |  |  |  |
| 11. SIAS | .66*** | -.22*** | .02 | -.20*** | -.66*** | -.13*** | -.13*** | -.03 | .81*** | .81*** | — |  |  |  |  |  |
| 12. Enjoyment | -.17*** | .28*** | .03 | .03 | .05* | .20*** | .17*** | .02 | .08* | -.09*** | -.16*** | — |  |  |  |  |
| 13. Laughter | -.35*** | .41*** | .06** | .02 | .46*** | .15*** | .04 | .04 | .23*** | -.32*** | -.15*** | .55*** | — |  |  |  |
| 14. VerbalHumor | -.30*** | .53*** | .13*** | -.08* | .37*** | .09*** | .04 | .13*** | .26*** | -.08* | -.15*** | .65*** | .48*** | — |  |  |
| 15. EverydayLifeHum | -.30*** | .47*** | .11*** | -.02 | .44*** | .13*** | .04* | .00 | .23*** | -.29*** | -.15*** | .73*** | .54*** | .65*** | — |  |
| 16. LaughSelf | -.45*** | .72*** | .09*** | -.09* | .39*** | .26*** | -.05* | .01 | .76*** | -.27*** | -.14*** | .68*** | .42*** | .55*** | .59*** | — |
| 17. HumStress | -.31*** | .51*** | .18*** | -.15*** | .38*** | .19*** | -.06 | .04 | .55*** | -.25*** | -.15*** | .73*** | .53*** | .66*** | .70*** | .73*** |

*Note*. Pearson’s *r* coefficients are reported. *p < .05, **p < .01, ***p < .001.
