# Supplementary material for: Who Laughs, and Who Doesn't? Predicting Humor Skills From Personality, Social Anxiety, and Laughter Dispositions of Gelotophobia, Gelotophilia, and Katagelasticism
Source: Scand J Psychol. 2026 May 20;67(6):1382–91. doi: 10.1111/sjop.70116 (PMC13739008; doi:10.1111/sjop.70116)
Supplement: Supplementary file 3 [file SJOP-67-1382-s003.docx]

# Supplementary Materials 2

# Table 1

Hierarchical Multiple Regression Predicting Laughter

| Model | Predictor | B | SE B | β | t | p | VIF |
| --- | --- | --- | --- | --- | --- | --- | --- |
| M₀ | Intercept | 5.50 | 0.44 | — | 12.41 | < .001 | — |
|  | Gender | -1.42×10⁻⁴ | 5.06×10⁻⁴ | -0.01 | -0.28 | .78 | 1.01 |
|  | Age | -0.03 | 0.02 | -0.05 | -1.42 | .16 | 1.01 |
| M₁ | Intercept | 2.69 | 0.51 | — | 5.29 | < .001 | — |
|  | Gender | -9.51×10⁻⁷ | 4.50×10⁻⁴ | -6.76×10⁻⁵ | -0.002 | 1.00 | 1.03 |
|  | Age | -0.03 | 0.02 | -0.05 | -1.54 | .12 | 1.02 |
|  | Honesty | -7.15×10⁻³ | 0.05 | -0.15 | 0.88 | .38 | 1.18 |
|  | Emotionality | 0.12 | 0.05 | 0.08 | 2.45 | .01 | 1.09 |
|  | Extraversion | 0.65 | 0.05 | 0.46 | 12.44 | < .001 | 1.06 |
|  | Agreeableness | 0.12 | 0.05 | 0.08 | 2.26 | .02 | 1.20 |
|  | Conscientiousness | -0.06 | 0.05 | -0.04 | -1.18 | .24 | 1.10 |
|  | Openness | 0.03 | 0.04 | 0.02 | 0.63 | .53 | 1.04 |
| M₂ | Intercept | 3.00 | 0.56 | — | 5.38 | < .001 | — |
|  | Gender | 1.47×10⁻⁵ | 4.51×10⁻⁴ | 1.04×10⁻³ | 0.03 | .97 | 1.03 |
|  | Age | -0.03 | 0.02 | -0.05 | -1.51 | .13 | 1.02 |
|  | Honesty | -0.01 | 0.05 | -0.07 | -0.21 | .83 | 1.19 |
|  | Emotionality | 0.13 | 0.05 | 0.09 | 2.58 | .01 | 1.19 |
|  | Extraversion | 0.59 | 0.06 | 0.43 | 10.02 | < .001 | 1.82 |
|  | Agreeableness | 0.12 | 0.05 | 0.08 | 2.27 | .02 | 1.20 |
|  | Conscientiousness | -0.06 | 0.05 | -0.04 | -1.30 | .20 | 1.12 |
|  | Openness | 0.03 | 0.04 | 0.02 | 0.60 | .55 | 1.04 |
|  | SPS | 0.02 | 0.06 | 0.01 | 0.27 | .79 | 3.03 |
|  | SIAS | -0.08 | 0.07 | -0.07 | -1.12 | .26 | 3.89 |
| M₃ | Intercept | 2.24 | 0.64 | — | 3.53 | < .001 | — |
|  | Gender | -1.76×10⁻⁴ | 4.31×10⁻⁴ | -0.01 | -0.41 | .68 | 1.03 |
|  | Age | -0.03 | 0.02 | -0.04 | -1.30 | .20 | 1.02 |
|  | Honesty | -0.02 | 0.05 | -0.01 | -0.40 | .69 | 1.32 |
|  | Emotionality | 0.16 | 0.05 | 0.11 | 3.30 | .001 | 1.31 |
|  | Extraversion | 0.42 | 0.06 | 0.30 | 6.77 | < .001 | 2.14 |
|  | Agreeableness | 0.07 | 0.05 | 0.04 | 1.30 | .19 | 1.27 |
|  | Conscientiousness | -5.63×10⁻³ | 0.05 | -0.03 | -0.12 | .91 | 1.17 |
|  | Openness | 0.05 | 0.04 | 0.04 | 1.16 | .25 | 1.06 |
|  | SPS | 0.05 | 0.06 | 0.05 | 0.84 | .40 | 3.36 |
|  | SIAS | -0.08 | 0.07 | -0.07 | -1.20 | .23 | 3.94 |
|  | Pho | -0.15 | 0.09 | -0.08 | -1.64 | .10 | 2.51 |
|  | Phi | 0.49 | 0.07 | 0.27 | 7.46 | < .001 | 1.47 |
|  | Kat | 0.03 | 0.07 | 0.01 | 0.38 | .71 | 1.70 |

# Table 2

Hierarchical Multiple Regression Predicting Laughing at Self

| Model | Predictor | B | SE B | β | t | p | VIF |
| --- | --- | --- | --- | --- | --- | --- | --- |
| M₀ | Intercept | 5.17 | 0.54 | — | 9.50 | < .001 | — |
|  | Gender | 8.46×10⁻⁴ | 6.21×10⁻⁴ | 0.05 | 1.36 | .17 | 1.01 |
|  | Age | -0.02 | 0.03 | -0.02 | -0.65 | .51 | 1.01 |
| M₁ | Intercept | 2.52 | 0.63 | — | 4.00 | < .001 | — |
|  | Gender | 6.90×10⁻⁴ | 5.57×10⁻⁴ | 0.04 | 1.24 | .22 | 1.03 |
|  | Age | -0.02 | 0.03 | -0.03 | -0.86 | .39 | 1.02 |
|  | Honesty | 0.16 | 0.06 | 0.10 | 2.79 | < .01 | 1.18 |
|  | Emotionality | -0.03 | 0.06 | -0.02 | -0.59 | .55 | 1.09 |
|  | Extraversion | 0.66 | 0.06 | 0.39 | 11.73 | < .001 | 1.06 |
|  | Agreeableness | 0.30 | 0.06 | 0.16 | 4.70 | < .001 | 1.20 |
|  | Conscientiousness | -0.20 | 0.06 | -0.11 | -3.31 | < .001 | 1.10 |
|  | Openness | -0.02 | 0.05 | -0.01 | -0.45 | .65 | 1.04 |
| M₂ | Intercept | 2.58 | 0.69 | — | 3.75 | < .001 | — |
|  | Gender | 7.29×10⁻⁴ | 5.59×10⁻⁴ | 0.04 | 1.30 | .19 | 1.03 |
|  | Age | -0.02 | 0.03 | -0.03 | -0.83 | .41 | 1.02 |
|  | Honesty | 0.16 | 0.06 | 0.09 | 2.67 | < .01 | 1.19 |
|  | Emotionality | -0.02 | 0.06 | -0.01 | -0.26 | .79 | 1.19 |
|  | Extraversion | 0.65 | 0.07 | 0.38 | 8.87 | < .001 | 1.82 |
|  | Agreeableness | 0.30 | 0.06 | 0.16 | 4.67 | < .001 | 1.20 |
|  | Conscientiousness | -0.21 | 0.06 | -0.12 | -3.39 | < .001 | 1.12 |
|  | Openness | -0.03 | 0.05 | -0.02 | -0.48 | .63 | 1.04 |
|  | SPS | -0.09 | 0.08 | -0.07 | -1.25 | .21 | 3.03 |
|  | SIAS | 0.06 | 0.09 | 0.04 | 0.69 | .49 | 3.90 |
| M₃ | Intercept | 1.46 | 0.60 | — | 2.43 | .02 | — |
|  | Gender | 1.75×10⁻⁴ | 4.07×10⁻⁴ | 0.01 | 0.43 | .67 | 1.03 |
|  | Age | -4.26×10⁻³ | 0.02 | -0.05 | -0.22 | .83 | 1.02 |
|  | Honesty | 0.09 | 0.05 | 0.05 | 2.01 | .05 | 1.33 |
|  | Emotionality | 0.06 | 0.05 | 0.03 | 1.29 | .20 | 1.31 |
|  | Extraversion | 0.13 | 0.06 | 0.08 | 2.29 | .02 | 2.15 |
|  | Agreeableness | 0.13 | 0.05 | 0.07 | 2.75 | < .01 | 1.27 |
|  | Conscientiousness | -0.07 | 0.05 | -0.04 | -1.54 | .12 | 1.17 |
|  | Openness | 0.02 | 0.04 | 0.01 | 0.59 | .55 | 1.06 |
|  | SPS | 0.04 | 0.06 | 0.03 | 0.65 | .51 | 3.37 |
|  | SIAS | 0.07 | 0.06 | 0.06 | 1.11 | .27 | 3.95 |
|  | Pho | -0.55 | 0.09 | -0.24 | -6.39 | < .001 | 2.52 |
|  | Phi | 1.37 | 0.06 | 0.66 | 22.33 | < .001 | 1.47 |
|  | Kat | -0.08 | 0.07 | -0.04 | -1.22 | .22 | 1.70 |

# Table 3

Hierarchical Multiple Regression Predicting Humor Under Stress

| Model | Predictor | B | SE B | β | t | p | VIF |
| --- | --- | --- | --- | --- | --- | --- | --- |
| M₀ | Intercept | 5.20 | 0.55 | — | 9.43 | < .001 | — |
|  | Gender | 2.76×10⁻⁴ | 6.29×10⁻⁴ | 0.02 | 0.44 | .66 | 1.01 |
|  | Age | -0.02 | 0.03 | -0.03 | -0.81 | .42 | 1.01 |
| M₁ | Intercept | 3.55 | 0.65 | — | 5.45 | < .001 | — |
|  | Gender | 4.04×10⁻⁵ | 5.76×10⁻⁴ | 2.31×10⁻³ | 0.07 | .94 | 1.03 |
|  | Age | -0.03 | 0.03 | -0.04 | -1.11 | .27 | 1.02 |
|  | Honesty | -0.06 | 0.06 | -0.03 | -0.93 | .35 | 1.18 |
|  | Emotionality | -0.15 | 0.06 | -0.08 | -2.40 | .02 | 1.09 |
|  | Extraversion | 0.63 | 0.06 | 0.37 | 10.90 | < .001 | 1.06 |
|  | Agreeableness | 0.25 | 0.07 | 0.14 | 3.86 | < .001 | 1.20 |
|  | Conscientiousness | -0.15 | 0.06 | -0.08 | -2.48 | .01 | 1.10 |
|  | Openness | 0.04 | 0.06 | 0.02 | 0.64 | .52 | 1.04 |
| M₂ | Intercept | 3.47 | 0.71 | — | 4.89 | < .001 | — |
|  | Gender | -2.97×10⁻⁵ | 5.75×10⁻⁴ | -1.70×10⁻³ | -0.05 | .96 | 1.03 |
|  | Age | -0.03 | 0.03 | -0.04 | -1.18 | .24 | 1.02 |
|  | Honesty | -0.04 | 0.06 | -0.03 | -0.74 | .46 | 1.19 |
|  | Emotionality | -0.18 | 0.06 | -0.10 | -2.84 | < .01 | 1.19 |
|  | Extraversion | 0.64 | 0.08 | 0.37 | 8.42 | < .001 | 1.82 |
|  | Agreeableness | 0.26 | 0.07 | 0.14 | 3.91 | < .001 | 1.20 |
|  | Conscientiousness | -0.14 | 0.06 | -0.08 | -2.30 | .02 | 1.12 |
|  | Openness | 0.04 | 0.06 | 0.02 | 0.70 | .49 | 1.04 |
|  | SPS | 0.18 | 0.08 | 0.13 | 2.30 | .02 | 3.03 |
|  | SIAS | -0.12 | 0.09 | -0.09 | -1.37 | .17 | 3.90 |
| M₃ | Intercept | 1.71 | 0.77 | — | 2.21 | .03 | — |
|  | Gender | -3.60×10⁻⁴ | 5.25×10⁻⁴ | -0.02 | -0.69 | .49 | 1.03 |
|  | Age | -0.02 | 0.03 | -0.03 | -0.88 | .38 | 1.02 |
|  | Honesty | -0.03 | 0.06 | -0.02 | -0.58 | .56 | 1.33 |
|  | Emotionality | -0.09 | 0.06 | -0.05 | -1.53 | .13 | 1.31 |
|  | Extraversion | 0.32 | 0.07 | 0.19 | 4.33 | < .001 | 2.15 |
|  | Agreeableness | 0.19 | 0.06 | 0.10 | 3.07 | < .01 | 1.27 |
|  | Conscientiousness | -0.03 | 0.06 | -0.02 | -0.57 | .57 | 1.17 |
|  | Openness | 0.09 | 0.05 | 0.07 | 1.70 | .09 | 1.06 |
|  | SPS | 0.23 | 0.07 | 0.17 | 3.13 | < .01 | 3.37 |
|  | SIAS | -0.13 | 0.08 | -0.09 | -1.59 | .11 | 3.95 |
|  | Pho | -0.26 | 0.11 | -0.11 | -2.32 | .02 | 2.52 |
|  | Phi | 0.81 | 0.08 | 0.37 | 10.19 | < .001 | 1.47 |
|  | Kat | 0.17 | 0.09 | 0.07 | 1.90 | .06 | 1.70 |

# Table 4

Hierarchical Multiple Regression Predicting Verbal Humor

| Model | Predictor | B | SE B | β | t | p | VIF |
| --- | --- | --- | --- | --- | --- | --- | --- |
| M₀ | Intercept | 5.29 | 0.50 | — | 10.49 | < .001 | — |
|  | Gender | 6.76×10⁻⁴ | 5.75×10⁻⁴ | 0.04 | 1.18 | .24 | 1.01 |
|  | Age | -0.02 | 0.03 | -0.02 | -0.63 | .53 | 1.01 |
| M₁ | Intercept | 3.21 | 0.60 | — | 5.31 | < .001 | — |
|  | Gender | 6.07×10⁻⁴ | 5.35×10⁻⁴ | 0.04 | 1.14 | .26 | 1.03 |
|  | Age | -0.03 | 0.03 | -0.03 | -0.99 | .32 | 1.02 |
|  | Honesty | -0.07 | 0.06 | -0.05 | -1.26 | .21 | 1.18 |
|  | Emotionality | -0.07 | 0.06 | -0.04 | -1.17 | .24 | 1.09 |
|  | Extraversion | 0.57 | 0.05 | 0.36 | 10.66 | < .001 | 1.06 |
|  | Agreeableness | 0.05 | 0.06 | 0.03 | 0.76 | .44 | 1.20 |
|  | Conscientiousness | 5.05×10⁻³ | 0.06 | 0.03 | 0.09 | .93 | 1.10 |
|  | Openness | 0.18 | 0.05 | 0.12 | 3.51 | < .001 | 1.04 |
| M₂ | Intercept | 3.31 | 0.66 | — | 5.01 | < .001 | — |
|  | Gender | 5.77×10⁻⁴ | 5.36×10⁻⁴ | 0.04 | 1.08 | .28 | 1.03 |
|  | Age | -0.03 | 0.03 | -0.03 | -1.01 | .31 | 1.02 |
|  | Honesty | -0.07 | 0.06 | -0.04 | -1.17 | .24 | 1.19 |
|  | Emotionality | -0.08 | 0.06 | -0.05 | -1.34 | .18 | 1.19 |
|  | Extraversion | 0.55 | 0.07 | 0.35 | 7.84 | < .001 | 1.82 |
|  | Agreeableness | 0.05 | 0.06 | 0.03 | 0.79 | .43 | 1.20 |
|  | Conscientiousness | 7.89×10⁻³ | 0.06 | 0.05 | 0.14 | .89 | 1.12 |
|  | Openness | 0.19 | 0.05 | 0.12 | 3.53 | < .001 | 1.04 |
|  | SPS | 0.10 | 0.07 | 0.08 | 1.15 | .30 | 3.03 |
|  | SIAS | -0.10 | 0.08 | -0.08 | -1.22 | .22 | 3.90 |
| M₃ | Intercept | 1.33 | 0.70 | — | 1.91 | .06 | — |
|  | Gender | 2.36×10⁻⁴ | 4.73×10⁻⁴ | 0.01 | 0.50 | .62 | 1.03 |
|  | Age | -0.02 | 0.02 | -0.02 | -0.71 | .48 | 1.02 |
|  | Honesty | -0.06 | 0.05 | -0.04 | -1.19 | .24 | 1.33 |
|  | Emotionality | -2.74×10⁻³ | 0.05 | -0.01 | -0.55 | .96 | 1.31 |
|  | Extraversion | 0.24 | 0.07 | 0.15 | 3.51 | < .001 | 2.15 |
|  | Agreeableness | -0.03 | 0.06 | -0.02 | -0.53 | .60 | 1.27 |
|  | Conscientiousness | 0.13 | 0.05 | 0.08 | 2.41 | .02 | 1.17 |
|  | Openness | 0.24 | 0.05 | 0.15 | 5.06 | < .001 | 1.06 |
|  | SPS | 0.14 | 0.07 | 0.11 | 2.09 | .04 | 3.37 |
|  | SIAS | -0.12 | 0.07 | -0.09 | -1.59 | .11 | 3.95 |
|  | Pho | -0.16 | 0.10 | -0.08 | -1.63 | .10 | 2.51 |
|  | Phi | 0.91 | 0.07 | 0.45 | 12.75 | < .001 | 1.47 |
|  | Kat | 0.11 | 0.08 | 0.05 | 1.40 | .16 | 1.70 |

# Table 5

Hierarchical Multiple Regression Predicting Everyday Humor

| Model | Predictor | B | SE B | β | t | p | VIF |
| --- | --- | --- | --- | --- | --- | --- | --- |
| M₀ | Intercept | 5.61 | 0.47 | — | 11.98 | < .001 | — |
|  | Gender | 3.13×10⁻⁴ | 5.34×10⁻⁴ | 0.02 | 0.59 | .56 | 1.01 |
|  | Age | -0.02 | 0.03 | -0.03 | -0.75 | .45 | 1.01 |
| M₁ | Intercept | 2.56 | 0.54 | — | 4.73 | < .001 | — |
|  | Gender | 3.24×10⁻⁴ | 4.78×10⁻⁴ | 0.02 | 0.68 | .50 | 1.03 |
|  | Age | -0.02 | 0.02 | -0.03 | -0.91 | .37 | 1.02 |
|  | Honesty | -0.05 | 0.05 | -0.04 | -1.06 | .29 | 1.18 |
|  | Emotionality | 0.04 | 0.05 | 0.03 | 0.84 | .40 | 1.09 |
|  | Extraversion | 0.63 | 0.05 | 0.43 | 13.14 | < .001 | 1.06 |
|  | Agreeableness | 0.17 | 0.05 | 0.11 | 3.08 | < .01 | 1.20 |
|  | Conscientiousness | 0.02 | 0.05 | 0.02 | 0.46 | .65 | 1.10 |
|  | Openness | 0.12 | 0.05 | 0.08 | 2.46 | .01 | 1.04 |
| M₂ | Intercept | 2.52 | 0.59 | — | 4.26 | < .001 | — |
|  | Gender | 3.25×10⁻⁴ | 4.79×10⁻⁴ | 0.02 | 0.68 | .50 | 1.03 |
|  | Age | -0.02 | 0.02 | -0.03 | -0.89 | .37 | 1.02 |
|  | Honesty | -0.05 | 0.05 | -0.04 | -1.05 | .29 | 1.19 |
|  | Emotionality | 0.04 | 0.05 | 0.03 | 0.80 | .42 | 1.19 |
|  | Extraversion | 0.64 | 0.06 | 0.43 | 10.12 | < .001 | 1.82 |
|  | Agreeableness | 0.17 | 0.05 | 0.11 | 3.08 | < .01 | 1.20 |
|  | Conscientiousness | 0.02 | 0.05 | 0.02 | 0.46 | .65 | 1.12 |
|  | Openness | 0.12 | 0.05 | 0.08 | 2.46 | .01 | 1.04 |
|  | SPS | -8.61×10⁻³ | 0.06 | -7.40×10⁻³ | -0.13 | .89 | 3.03 |
|  | SIAS | 0.01 | 0.07 | 0.01 | 0.01 | .85 | 3.90 |
| M₃ | Intercept | 1.78 | 0.65 | — | 2.73 | < .01 | — |
|  | Gender | 5.21×10⁻⁵ | 4.42×10⁻⁴ | 3.51×10⁻³ | 0.12 | .91 | 1.03 |
|  | Age | -0.01 | 0.02 | -0.02 | -0.57 | .57 | 1.02 |
|  | Honesty | -0.08 | 0.05 | -0.05 | -1.54 | .12 | 1.33 |
|  | Emotionality | 0.09 | 0.05 | 0.06 | 1.75 | .08 | 1.31 |
|  | Extraversion | 0.38 | 0.06 | 0.26 | 6.05 | < .001 | 2.15 |
|  | Agreeableness | 0.09 | 0.05 | 0.06 | 1.80 | .07 | 1.27 |
|  | Conscientiousness | 0.10 | 0.05 | 0.06 | 1.96 | .05 | 1.17 |
|  | Openness | 0.14 | 0.04 | 0.10 | 3.30 | < .01 | 1.06 |
|  | SPS | 0.05 | 0.06 | 0.05 | 0.86 | .39 | 3.37 |
|  | SIAS | 0.02 | 0.07 | 0.01 | 0.25 | .80 | 3.95 |
|  | Pho | -0.27 | 0.09 | -0.13 | -2.86 | < .01 | 2.52 |
|  | Phi | 0.67 | 0.07 | 0.36 | 10.00 | < .001 | 1.47 |
|  | Kat | 6.23×10⁻³ | 0.07 | 3.22×10⁻³ | 0.08 | .93 | 1.70 |

# Table 6

Hierarchical Multiple Regression Predicting Humor Enjoyment

| Model | Predictor | B | SE B | β | t | p |
| --- | --- | --- | --- | --- | --- | --- |
| M₀ | Intercept | 5.05 | 0.45 | — | 11.14 | < .001 |
|  | Gender | 1.97×10⁻⁴ | 5.17×10⁻⁴ | 0.01 | 0.38 | .70 |
|  | Age | -0.01 | 0.02 | -0.01 | -0.42 | .68 |
| M₁ | Intercept | 2.95 | 0.57 | — | 5.15 | < .001 |
|  | Gender | 1.72×10⁻⁴ | 5.05×10⁻⁴ | 0.01 | 0.34 | .73 |
|  | Age | -8.69×10⁻³ | 0.02 | -0.01 | -0.36 | .72 |
|  | Honesty | -0.03 | 0.05 | -0.02 | -0.51 | .61 |
|  | Emotionality | 0.13 | 0.05 | 0.09 | 2.52 | .01 |
|  | Extraversion | 0.27 | 0.05 | 0.19 | 5.36 | < .001 |
|  | Agreeableness | 0.22 | 0.06 | 0.14 | 3.71 | < .001 |
|  | Conscientiousness | -0.05 | 0.05 | -0.03 | -0.83 | .41 |
|  | Openness | 0.08 | 0.05 | 0.06 | 1.63 | .10 |
| M₂ | Intercept | 3.31 | 0.62 | — | 5.30 | < .001 |
|  | Gender | 1.66×10⁻⁴ | 5.05×10⁻⁴ | 0.01 | 0.33 | .74 |
|  | Age | -8.62×10⁻³ | 0.02 | -0.01 | -0.36 | .72 |
|  | Honesty | -0.03 | 0.05 | -0.02 | -0.50 | .62 |
|  | Emotionality | 0.14 | 0.06 | 0.09 | 2.45 | .01 |
|  | Extraversion | 0.21 | 0.07 | 0.15 | 3.13 | < .01 |
|  | Agreeableness | 0.22 | 0.06 | 0.14 | 3.74 | < .001 |
|  | Conscientiousness | -0.05 | 0.05 | -0.03 | -0.90 | .37 |
|  | Openness | 0.08 | 0.05 | 0.06 | 1.62 | .10 |
|  | SPS | 0.09 | 0.07 | 0.08 | 1.27 | .20 |
|  | SIAS | -0.14 | 0.08 | -0.13 | -1.86 | .06 |
| M₃ | Intercept | 2.29 | 0.73 | — | 3.15 | < .01 |
|  | Gender | 1.47×10⁻⁵ | 4.94×10⁻⁴ | 1.02×10⁻³ | 0.03 | .98 |
|  | Age | -4.18×10⁻³ | 0.02 | -6.08×10⁻³ | -0.18 | .86 |
|  | Honesty | -0.02 | 0.06 | -0.01 | -0.38 | .71 |
|  | Emotionality | 0.17 | 0.06 | 0.12 | 3.04 | < .01 |
|  | Extraversion | 0.07 | 0.07 | 0.05 | 0.97 | .33 |
|  | Agreeableness | 0.18 | 0.06 | 0.12 | 3.14 | < .01 |
|  | Conscientiousness | 8.48×10⁻³ | 0.05 | 0.06 | 1.15 | .88 |
|  | Openness | 0.11 | 0.05 | 0.07 | 2.15 | .03 |
|  | SPS | 0.10 | 0.07 | 0.09 | 1.41 | .16 |
|  | SIAS | -0.15 | 0.08 | -0.14 | -2.02 | .04 |
|  | Pho | -0.05 | 0.11 | -0.03 | -0.51 | .61 |
|  | Phi | 0.41 | 0.07 | 0.23 | 5.55 | < .001 |
|  | Kat | 0.06 | 0.08 | 0.03 | 0.79 | .43 |
